# GSK3: A Kinase Balancing Promotion and Resolution of Inflammation

**DOI:** 10.3390/cells9040820

**Published:** 2020-03-28

**Authors:** Leonie Hoffmeister, Mareike Diekmann, Korbinian Brand, René Huber

**Affiliations:** Institute of Clinical Chemistry, Hannover Medical School, 30625 Hannover, Germany; hoffmeister.leonie@mh-hannover.de (L.H.); diekmann.mareike@mh-hannover.de (M.D.); brand.korbinian@mh-hannover.de (K.B.)

**Keywords:** GSK3α, GSK3β, inflammation, resolution of inflammation, infection, NF-κB, AP-1, C/EBP

## Abstract

GSK3 has been implicated for years in the regulation of inflammation and addressed in a plethora of scientific reports using a variety of experimental (disease) models and approaches. However, the specific role of GSK3 in the inflammatory process is still not fully understood and controversially discussed. Following a detailed overview of structure, function, and various regulatory levels, this review focusses on the immunoregulatory functions of GSK3, including the current knowledge obtained from animal models. Its impact on pro-inflammatory cytokine/chemokine profiles, bacterial/viral infections, and the modulation of associated pro-inflammatory transcriptional and signaling pathways is discussed. Moreover, GSK3 contributes to the resolution of inflammation on multiple levels, e.g., via the regulation of pro-resolving mediators, the clearance of apoptotic immune cells, and tissue repair processes. The influence of GSK3 on the development of different forms of stimulation tolerance is also addressed. Collectively, the role of GSK3 as a kinase balancing the initiation/perpetuation and the amelioration/resolution of inflammation is highlighted.

## 1. Introduction

Glycogen synthase kinase (GSK) 3 is a serine/threonine kinase consisting of two highly similar paralogs, GSK3α and β. With more than 40 definitely identified targets and more than 500 proposed candidate substrates, GSK3 is involved in the regulation of a plethora of intracellular/molecular, organic/cellular, and (patho-)physiological events [1]. For instance, GSK3 has an influence on transcriptional regulation, alternative splicing [2,3], and mRNA stability [4]. On the protein level, GSK3 affects translation and protein synthesis [5,6], protein activity, localization, and degradation [7] (including its participation in the β-catenin destruction complex [8]). Metabolic processes, such as glucose metabolism [7], lipid deposition [9] and accumulation [10], and processes associated with mitochondrial activity (biogenesis, bioenergetics, permeability, and motility [11]), are influenced by GSK3. In consequence, relevant cellular functions are governed by GSK3. These include proliferation, differentiation, apoptosis, [1], adhesion, and migration [12]. Amongst others, GSK3 is of importance for physiological processes, such as embryonic and tissue development [1,7], tumor suppression [13], innate and adaptive immune responses [14], a variety of neuronal functions, and circadian rhythm regulation [12].

GSK3 also plays a role in the pathogenesis of common diseases, e.g., neurological/neurodegenerative diseases [13,15]; diabetes mellitus [16]; inflammatory diseases [17], such as rheumatoid arthritis [18]; and different types of cancer [19]. Moreover, GSK3 is also of interest in geriatrics [15,20]. Thus, the modulation of GSK3 (especially GSK3β) activity via natural compounds [21] or the design of pharmacologically applicable inhibitors [19] is still a promising target for various therapeutic approaches.

Since the general aspects of GSK3 functions have been extensively reviewed elsewhere (e.g., [6,7,13,22] and others) they shall not be the topic of this review. Following the description of the basic molecular and mechanistic features of GSK3, we will focus on the janiform nature of GSK3 within the regulation of both the promotion and the termination of inflammation.

## 2. Structure and Function of GSK3

### 2.1. Protein Structure, Enzymatic Activity, and Substrate Specificity

#### 2.1.1. General Aspects

GSK3 protein was initially purified in 1980 from rabbit skeletal muscle and characterized as an enzyme activating adenosine triphosphate/magnesium-dependent protein phosphatase [23,24] and phosphorylating glycogen synthase (GS) [25,26], thus inhibiting its activity and negatively controlling the final step of glycogen synthesis [6]. In 1990, nucleotide sequence analysis of a rat brain cDNA library revealed the existence of two highly similar GSK3 paralogs termed α and β [27] and further analyses led to the identification of two GSK3β splice variants (GSK3β1 and 2; see Section 2.2.1.) [28]. Due to their remarkable homology (see Section 2.2.1.), most GSK3-targeting small molecule inhibitors address the ATP binding pocket of both GSK3α and β, rendering their selective inhibition a persistent challenge [12]. However, due to the presence of a single amino acid (aa) exchange between α and β in the ATP binding pocket within the kinase domain (GSK3α-Glu196 → GSK3β-Asp133), the development of paralog-selective inhibitors has been reported recently (GSK3α: BRD0705, GSK3β: BRD3731) [29]. Though highly similar, GSK3α and β are differentially regulated and expressed, exhibit tissue-specific expression profiles/levels (see Section 2.2.), have common as well as distinct substrates and cellular functions, and cannot replace each other completely [12]. Moreover, the loss of one paralog does not result in an enhanced expression or activity of the remaining gene/protein [30].

#### 2.1.2. Structure and Molecular Function

GSK3 is a monomeric serine/threonine kinase belonging to the CMGC kinase group (named after the founding kinase families cyclin-dependent kinases, mitogen-activated protein kinases (MAPK), GSK, and CDC-like kinases) [31]. GSK3β contains a negative regulatory N-terminal domain, the kinase domain including both the ATP binding site and the enzymatically active site, as well as a C-terminal domain also possessing negative regulatory capacity (for details, see Section 2.2.3.). GSK3α, basically characterized by the same protein structure, contains an extended and glycine-rich N-terminal domain [32,33]. Within the active site, conserved Lys residues (GSK3α: Lys148 and 149; GSK3β: Lys85 and 86) are responsible for ATP binding and catalyzing the γ-phosphate transfer to the substrate; thus, local point mutations (GSK3α: Lys148Arg; GSK3β: Lys85Arg) have a dominant-negative effect on GSK3 enzymatic activity [34]. 

GSK3 generally targets the consensus aa sequence Ser/Thr_1_-X_1_-X_2_-X_3_-Ser/Thr_2_. Though not absolutely required, X_1_ is often a proline [35]. In dependency of prior phosphorylation (“priming”) of the C-terminal serine/threonine residue (Ser/Thr_2_), the primed substrate is able to associate with the positively charged GSK3 substrate binding domain, thus enabling the phosphorylation of the respective substrate at the N-terminal serine or threonine (Ser/Thr_1_) [12,13,36] with a massively increased efficiency (up to 1000-fold) in comparison to unprimed substrates [32,37]. Though the number of intervening residues is generally three in most substrates, deviating numbers have been described [13]. Moreover, in GSK3 substrates that are subsequently addressed by β-TrCP (“F-box containing E3 ligase β-transducin repeat-containing protein”) for ubiquitin-mediated degradation, a more specific GSK3 recognition sequence, i.e., Asp-Ser-Gly-X-X-Ser, is present [7]. While the initial priming phosphorylation has to be mediated by another kinase, GSK3 is able to perform multiple successive phosphorylation steps at substrates containing a series of adjacent consensus sequences, thus generating its own priming phosphorylation for further phosphorylation events [38]. Only a limited number of substrates (e.g., Jun, avian myelocytomatosis viral oncogene homolog (Myc), histone H1.5, microtubule affinity-regulating kinase 2, CCAAT/enhancer binding protein (C/EBP) β, Tau protein, and p21) may be bound and phosphorylated at specific sites without priming phosphorylation [7,12,39], potentially due to the presence of acidic residues in the C-terminal position [13]. However, this assumption is still controversially discussed [7] and an influence of protein conformation has also been proposed [12]. In addition, other motifs in these proteins may be addressed by GSK3 via the conservative mechanism, as shown for Jun and Tau following Jun N-terminal kinase (JNK)-mediated priming phosphorylation [35] and C/EBPβ after extracellular signal-regulated kinase (ERK) 2-mediated priming [40].

### 2.2. Regulation of GSK3

#### 2.2.1. GSK3 Gene Structure and mRNA Expression

Neither the GSK3α nor the GSK3β promoter contains a classical TATA box and in contrast to GSK3α, GSK3β also misses an initiator element [41,42]. Both genes are regulated by a variety of common transcription factors (TFs), such as activator protein (AP-)1, specificity protein 1, cyclic adenosine monophosphate (cAMP-)responsive element binding protein (CREB), and myeloid zinc finger 1 [41,42,43]. The GSK3α promoter additionally provides binding sites for late SV40 factor and Yin and Yang 1 [42], whilst GSK3β enables the binding of the TF Tst-1 (“Tuberculin skin test reactivity, absence of”), Myb (“myoblastosis”), TFCP2, and AP-2 [41]. The GSK3β promoter further possesses several CCAAT boxes and inverted CCAAT elements [41] that are recognized by members of the C/EBP family, such as C/EBPα and β [44]. Another binding site for TF AP-4 is generated by the minor allele of a single nucleotide polymorphism (SNP; rs334,558) in the GSK3β promoter [45].

Both genes possess approximately 85% overall sequence identity, including 98% identity within the catalytic domain. The C-terminal section is more variable, showing only 36% homology [27,32]. In addition, GSK3α and β are widely conserved among a variety of different species [46], with the exception of birds, which proved to be naturally occurring GSK3α knock-out (KO) organisms [47]. The human GSK3α gene (GSK3A, chromosomal localization: 19q13.2) is expressed as a single mRNA comprising 11 exons with 2193 bases (gene bank accession number: NM_019884.3; average G/C content: 61%), including a 5′ untranslated region (UTR) of 137 bases (83% G/C), the protein coding sequence (1452 bases, including stop codon; 61% G/C), and a 3′UTR of 604 bases (58% G/C) [48]. Human GSK3β (GSK3B, 3q13.33) is encoded by a total of 12 exons, yielding three variants comprising 7134 (NM_002093.3, transcript variant 1), 7095 (NM_001146156.1, transcript variant 2), and 6982 bases (NM_001354596.1, transcript variant 3). Each variant (42% GC) includes a 5′UTR of 983 bases (58% G/C), the protein coding sequence (1302 bases for variants 1 and 3, 1263 bases for variant 2 lacking exon 9; 47% G/C), and a longer 3’UTR (4849 bases for variants 1 and 2, 4697 bases for variant 3; 37% G/C) [48,49]. Generation of the different variants depends on RNA helicases DEAD-box 5- and 17-controlled [50] alternative splicing [51] that may be influenced by the occurrence of intronic SNPs [45]. In other organisms, however, divergent numbers of variants have been described, e.g., five differentially expressed porcine GSK3β isoforms [52] and six transcripts in the goat [53].

GSK3α mRNA and protein as well as GSK3β mRNA are ubiquitously but variably expressed, mostly at medium to high levels, in all human tissues and organs. GSK3β protein expression, however, is slightly more restricted; can hardly be detected in human heart, skeletal, and smooth muscles as well as in lung, vagina, and soft tissues; and is most strongly expressed in the brain and neuronal tissue [54]. Moreover, in many tissues, a remarkable discrepancy between mRNA and protein levels can be observed [46,54], an effect that may be ascribed to differentially regulated and paralog-specific transcriptional and translational events [46]. However, in GSK3-expressing cells/tissues, its expression levels remain relatively stable and only a limited number of cellular conditions appear to exist in which GSK3 expression is significantly regulated [13], including murine brain development [55] and maturation of murine Th17 lymphocytes [56]. GSK3 mRNA amounts may be adjusted by mRNA stability-modifying proteins, such as human antigen R [57].

#### 2.2.2. GSK3 Proteins—Paralogs, Isoforms, Expression, and Localization

Translation of the human GSK3 mRNAs results in the synthesis of one GSK3α (protein bank accession number: NP_063937.2) and two GSK3β protein isoforms, i.e., GSK3β1 (NP_001139628.1, derived from transcript variant 2) and GSK3β2 (NP_002084.2, derived from transcript variants 1 and 3). GSK3α is a 51 kDa protein consisting of 483 aa. Both GSK3β isoforms are slightly shorter (GSK3β1: 420 aa, approximately 47 kDa; GSK3β2: 433 aa, approximately 49 kDa) [12,39,58]. GSK3β1 is the ubiquitously expressed isoform, whereas the longer GSK3β2 variant (first identified in 2002 in the rat brain [28]) possesses an additional external loop of 13 aa located within the catalytic domain between residues 303 and 304 [12,59], exhibits differential substrate specificity and efficiency in comparison to GSK3β1 [60], and appears to be predominately expressed in neurons and involved in neuronal functions [12,61,62].

GSK3 paralogs show distinct patterns of intracellular distribution [12]. GSK3α, though in principle capable of nuclear translocation, is virtually exclusively located in the cytosol due to its fast export from the nucleus [63]. GSK3β is also mainly a cytoplasmic protein but nonetheless well detectable in both the nucleus and mitochondria [64] and at further membrane compartments, such as endosomes and lysosomes [65]. However, an aberrant accumulation of active GSK3β has been described in several cancer cells [19]. A 19 aa comprising nuclear localization sequence (NLS) has been described to be present in a basic loop (aa 85–123) within the kinase domain. To ensure cytoplasmic localization, the NLS is sequestered by cytosolic protein complexes [66]. Interestingly, the amount of active GSK3β is significantly higher in the extra-cytoplasmic areas (5- to 8-fold) [64]. Intracellular localization is controlled at different regulatory levels [12]. These include (de-)phosphorylation [67], association with proteins facilitating nuclear import (e.g., karyopherin β2 [68] or herpesvirus-derived latency-associated nuclear antigen (LANA) [69]) or export (e.g., GSK3-binding protein [70]), and partial digestion (see Section 2.2.3.). Recently, it has been shown that inactivation of the phosphatidylinositol-3-kinase (PI3K), protein kinase B (PKB/Akt), mechanistic target of rapamycin complex (mTORC) 1 axis results in the nuclear accumulation of both GSK3α and β. In contrast, active PI3K-Akt-mTORC1 favors the retention of GSK3β in the cytosol, including (partial) localization at endomembrane compartments [65].

#### 2.2.3. Posttranslational Modifications

##### Phosphorylation

GSK3 shows a constitutively high basal activity in resting cells, which can be affected by post-translational modifications [12,13]. The presence of an activating phosphorylation at Tyr279 (GSK3α) and Tyr216 (GSK3β) is assumed to be necessary for proper formation of the catalytic domain during protein folding [39] and has been demonstrated to significantly facilitate substrate accessibility [71], to increase GSK3 protein stability [72], and to be essential for maximum enzymatic activity [73,74], thus increasing its catalytic capacity (approximately 5-fold, [75]). However, replacing this tyrosine by phenylalanine via in vitro mutagenesis had only little effects on GSK3 activity [34,75,76], indicating that Tyr279/216 phosphorylation is not an indispensable prerequisite for basal enzymatic activity. Moreover, these sites are prone to auto-phosphorylation [72,77] and are already phosphorylated following translation during heat shock protein 90 (Hsp90)-dependent protein folding, thus directly yielding active enzymes [13,78]. The tyrosine protein kinases src, fyn, and Pyk2 may further contribute to Tyr279/216 phosphorylation [79]. Though Tyr279/216 phosphorylation proved to be very stable [72], it can be dynamically regulated under certain conditions as demonstrated in models of neurodegeneration [7,80]. Decreased GSK3 activity is associated with reduced tyrosine phosphorylation under various conditions and thus, a regulatory role for phospho-tyrosine phosphatases has been proposed [13].

However, the major regulatory influence on GSK3 is mediated via inhibitory events, mainly in response to a variety of stimulating agents (e.g., insulin, serum, growth factors), leading to the fast reduction of enzymatic activity (30%–70% within 10 min) [12]. At the molecular level, this is predominantly mediated via inhibitory phosphorylation at different residues mainly located in the N-terminal region [79]. Among these, Ser21 and Ser9 are regarded as the most important regulatory sites in GSK3α and β [39,79] due to the function of the N-terminal protein domain as a pseudo-substrate [6]. This feature results from the interaction of phosphorylated Ser21/9 with three highly conserved basic residues [81] located within the priming phosphate binding site (GSK3α: Arg159, Arg243, and Lys268; GSK3β: Arg96, Arg180, and Lys205 [34,71,74,82]) blocking both the primed substrate binding and the catalytic domain [6]. Thus, Ser21/9 phosphorylation is sufficient for overriding Tyr279/216 phosphorylation-dependent GSK3 activation [80]. This mechanism also opens the possibility that phosphorylation of the few unprimed GSK3 substrates may not be negatively affected by Ser21/9 phosphorylation [39].

In GSK3β, activating effects of Ser147 phosphorylation [83] and inhibitory effects of phosphorylated Thr43 (acting as a priming phosphorylation for Ser9-dependent inactivation [84]) as well as Ser389 and Thr390 have also been observed [39,79]. In mice, an additional dual-specificity tyrosine phosphorylation-regulated kinase 1A-mediated inhibitory phosphorylation at Thr356 has been described [85], which still has to be established in humans. The respective sites are targeted by several kinases upon stimulation with multiple factors [39,79]. Ser21 and/or Ser9, for instance, can be phosphorylated by protein kinases A (PKA) in response to cAMP-inducing agents (e.g., forskolin or isoproterenol) and cAMP analogues [86], PKB/Akt (induced by tumor necrosis factor (TNF) [87], interferon (IFN) β [88], growth factors, insulin [89], and lipopolysaccharide (LPS) [90]), and various protein kinase C (PKC) isoforms (including α, βII, γ, δ, and η [91], e.g., induced by TNF [92], phorbol esters, and via the Wnt (“wingless-type MMTV integration site family member”) pathway [93]). Moreover, these residues may be triggered by p38-MAPK (in response to IL-13) [94], serum- and glucocorticoid-induced protein kinase [95], and both subfamilies of ribosomal protein S6 kinase (p90-RSK and p70-S6K, induced by growth factors) [39]. Interestingly, a Ser21/9 phosphorylation-independent mechanism of GSK3 inactivation in response to Wnt signaling has also been described [96], presumably involving GSK3 localization and restriction of substrate accessibility [7,97]. GSK3β-Ser147 phosphorylation is induced by Wnt signaling via PKCζ [83], Thr43 phosphorylation by growth factors via ERK [84], and Thr390 in response to cellular stress or Wnt proteins via p38 [98].

Among the known growth factors, insulin-like growth factor 1 [99], platelet-derived growth factor BB [100], hepatocyte growth factor [101], fibroblast growth factor 2 [102], epidermal growth factor, and transforming growth factor (TGF-)β [103] as well as stem cell factor [104] have been demonstrated to inhibit GSK3 activity. In turn, activation of Ser21/9-phosphorylated GSK3 can be mediated via protein phosphatases (PP) 1, 2A [79,105], and 2B [15].

##### Acetylation, Ribosylation, SUMOylation, Citrullination, Ubiquitination, and Methylation

Additional post-translational modifications may further modify GSK3 functions and activity [1,13]. Lysine acetylation has been shown to have a negative regulatory influence on GSK3 kinase activity in general. For instance, acetylation of GSK3α-Lys246 and GSK3β-Lys183 appears to hinder ATP binding [106]. Accordingly, deacetylases of the sirtuin (SIRT) family contribute to the activation of GSK3β, e.g., via deacetylation of Lys183 by SIRT2 [106] or Lys205 by SIRT1 [107] and 3 [108]. ADP-ribosyltransferase ARTD10-mediated mono-ADP-ribosylation also leads to an inhibition of GSK3β activity [109], whereas removal of ADP-ribose by mono-ADP-ribosylhydrolase macrodomain protein MacroD2 restores its activity [110]. GSK3β Lys292-SUMOylation appears to be required for its stability, kinase activity, and nuclear localization [111] and peptidyl-deiminase 4-mediated citrullination also supports its nuclear translocation [112]. Using high-throughput approaches, further post-translational modifications have been found in both GSK3α and GSK3β, including ubiquitination at multiple sites [113] and methylation of GSK3α-Arg16 [114] or GSK3β-Lys27 [115].

##### Proteolytic Cleavage and Degradation

Under certain conditions, GSK3 activity can be enhanced by proteolytic cleavage of its N- and/or C-terminus [116,117]. The inhibitory effects of Ser21/Ser9 phosphorylation, for instance, can be countered by matrix metalloprotease (MMP-)2 [118] or calpain-driven N-terminal cleavage of GSK3α and β, generating remarkably truncated but enzymatically active protein variants (MMP-2: GSK3β: 30 kDa; calpain: GSK3α: 42 and 30 kDa, GSK3β: 40 and 30 kDa) [116]. Further analyses revealed that residues Thr38-Thr39 and Ile384-Gln385 are the relevant calpain cleavage sites in GSK3β resulting in the formation of the fragments ΔN-GSK3β (aa 39–420), ΔC-GSK3β (aa 1–384), and ΔN/ΔC-GSK3β (aa 39–384, i.e., the most active form). The remaining (non-catalytic) C-terminus, however, appears to be essential for GSK3 enzymatic activity, since it was demonstrated in deletion experiments that the absence of aa 417–430 in GSK3α or aa 345–367 in GSK3β yields a virtually complete loss of both Tyr279/216 auto-phosphorylation and substrate (e.g., Tau) phosphorylation [34].

Truncation is facilitated by dephosphorylation of Ser9 (mainly via phosphatase 1/2A; both N- and C-terminal) and Ser389 (only C-terminal) [117]. C-terminal GSK3β truncation, in turn, enhances its nuclear translocation and association with PP2A, thus promoting its activation via Ser9 dephosphorylation [119]. In the case of GSK3α, the N-terminal region further appears to be responsible for its fast and efficient export from the nucleus, since N-terminal binding of specific calcium/calpain-sensitive interaction partner(s) or deletion of the N-terminus resulted in nuclear accumulation of GSK3α [63]. For GSK3β, it has been shown that the presence of the N-terminal part is necessary for the interaction with certain proteins, e.g., PKB/Akt, p53, or the 14–3-3ζ adapter protein [120].

Degradation of GSK3 proteins is predominantly mediated via polyubiquitination and subsequent proteasomal destruction in a Ser9 phosphorylation-dependent manner [95].

## 3. Role of GSK3 in Inflammation and the Resolution of Inflammation

In this chapter, the contribution of GSK3 to the steering of both the initiation/maintenance of inflammation and the resolution of inflammation will be highlighted.

### 3.1. GSK3 and Inflammation

#### 3.1.1. Cytokine Expression

GSK3 acts a potent driver of inflammation, rendering GSK3 inhibitors a promising target of anti-inflammatory research [17,121]. It is well established that enzymatically active GSK3 acts as a crucial positive regulator of pro-inflammatory cytokines (e.g., TNF, interleukin (IL-)1β, IL-6 [15], IL-17, IL-18 [122], IL-23 [94], IL-12, IFN-γ [17]), chemokines (IL-8 [122], C-C motif chemokine ligand (CCL) 2 [17], 3, 4 [123], and 12, C-X-C motif chemokine ligand (CXCL) 1, 2, 5 [17], and 10 [124]), and further pro-inflammatory mediators (e.g., nitric oxide (NO) [125] or prostaglandin E2 [126]). Vice versa, anti-inflammatory cytokines, such as IL-2 [127], IL-10 [90], IL-22 [128], IL-33 [129], and IL-1 receptor antagonist [130], are negatively regulated by GSK3. Interestingly, GSK3 has also been linked to anti-inflammatory action leading to the termination of inflammatory events (see Section 3.2.). As a reflection of the impact of GSK3β on inflammatory diseases, the application of the GSK3β activity index (i.e., the ratio of total to Ser9-phosphorylated GSK3β) has been proposed as a new diagnostic and prediction tool [131,132]. 

#### 3.1.2. Animal Models

The role of GSK3 has been assessed in numerous animal (especially mouse) KO models, including both conventional and conditional approaches [12,20]. In addition, in a variety of inflammatory animal disease models, the influence of GSK3 has been studied [121].

##### GSK3 KO Models

GSK3α KO mice were viable, fertile, had normal body mass [133], and no obvious skeletal defects [134], but showed increased water uptake and urine production [135], enhanced sensitivity towards insulin and glucose, reduced fat mass, and increased hepatic glycogen levels [133]. In an alternative GSK3α KO model, mice were also viable but characterized by higher body weight, heavier organs (especially brain, heart, and testis), male infertility, and slightly reduced lifespan [136]. Interestingly, despite a multitude of indications that GSK3 has an essential influence on inflammatory processes [17,121], GSK3α KO organisms are not characterized by significant signs of altered immune reactions, implying that GSK3β may be the more prominent paralog with respect to immune regulation.

In contrast to GSK3α, GSK3β KO proved to be lethal in the late phase of murine embryogenesis [12]. The respective mice are characterized by a variety of cellular and organ defects, including liver degeneration induced by TNF-hypersensitivity-related apoptosis of hepatocytes [137] and severe cardiac defects resulting from impaired cardiomyocyte differentiation [138]. Since the premature death of KO individuals impedes the analysis of GSK3β functions, several conditional, tissue-specific GSK3β KO models have been generated. Though not predominantly characterized by alterations of the immune system or in host defense, some aspects of conditional KO may also have an impact on inflammatory processes (e.g., proliferation, differentiation, apoptosis, metabolism, or TF activation). A myeloid cell-specific GSK3β KO, for instance, led to liver tissue/hepatocellular protection as well as diminished pro- (TNF, IL-6, CXCL10, neutrophil infiltration/activation) and enhanced anti-inflammatory responses (IL-10) in a murine model of ischemia-reperfusion injuries [139]. GSK3β KO in the renal proximal tubule proved to be protective against mortality and tubular injury due to rapid tissue regeneration as reflected by reduced apoptosis in the renal cortex and accelerated proliferation of renal proximal tubule cells in a mercury chloride-induced acute nephrotoxic injury model [140]. In murine experimental adriamycin nephropathy [141] and oxidative glomerular injury [142], podocyte-specific GSK3β KO significantly decreased podocyte loss and injury, reduced glomerular damage, attenuated proteinuria [141,142], and reduced glomerular reactive oxygen species (ROS) production, the latter presumably due to an increase in the antioxidant genes, inducing nuclear factor erythroid 2-related factor 2 [142,143]. The respective podocytes were characterized by increased glycogen accumulation [142] as well as preserved cytoskeleton integrity and focal adhesions, reduced mitochondria dysfunction, and diminished pro-inflammatory nuclear factor (NF-)κB activation [141]. Anti-apoptotic effects of GSK3β deficiency have also been observed in murine GSK3β KO oligodendrocytes that are protected from caspase-dependent (but not -independent) apoptosis [144]. Moreover, in heterozygous GSK3β^+/-^ mice exhibiting Pam3CSK4-induced peritonitis, the extent of inflammation was significantly lower than in the wildtype and equivalent results have been obtained in chimeric mice possessing GSK3β KO hematopoietic cells [145]. In summary, these data indicate that GSK3β indeed plays an essential role in controlling cellular functions contributing to initiation or resolution of inflammation (see also Section 3.2.).

##### Animal Models of Inflammatory Diseases

The influence of GSK3 on inflammatory events has also been studied in a number of animal models focusing on inflammatory diseases. For instance, in a murine collagen-induced arthritis (CIA) model, treatment with the GSK3 inhibitor TDZD-8 led to significantly reduced joint inflammation and destruction as reflected by reduced edema formation and histological joint alterations/bone resorption, decreased numbers of circulating leukocytes and infiltrating neutrophils as well as lower levels of TNF, IL-6, macrophage inflammatory protein (MIP) 1α and 2, inducible NO synthase (iNOS), and cyclooxygenase-2 (COX-2) [146]. In another murine CIA model, GSK3 inhibitors reduced hind paw erythema and swelling, pannus formation, infiltration of macrophages and T-cells, and bone erosion. Inhibitor-treated CIA mice also showed decreased pro-inflammatory cytokine production, e.g., TNF, IL-1β, IL-6, and IFN-γ [147]. In a collagen antibody-induced arthritis model, GSK3 inhibition using LiCl also reduced joint swelling [145]. Likewise, treatment with LiCl resulted in an amelioration of peritonitis in a murine Pam3CSK4-induced peritonitis model [145]. In mice with peptidoglycan (PGN)-induced peritonitis, anti-inflammatory effects (increased IL-10, decreased TNF, IL-1β, IL-6) could be elicited by ephedrine hydrochloride (EH), a substance that inhibits GSK3β via the PI3K-Akt axis in cell culture experiments [148] (see Section 3.1.3.). In a rat model of ischemic stroke, the application of GSK3 inhibitor VIII significantly reduced infarct volume, brain swelling, and neutrophil infiltration as well as signs of apoptosis (caspase-3 activation) and inflammation (COX-2 amounts; number of astrocytes, monocytes, and macrophages) [149]. In comparison to WT mice, protein tyrosine phosphatase receptor type O (PTPRO) KO mice are characterized by significantly attenuated inflammation (i.e., decreased amounts of TNF, IL-1β, IL-6, IFN-γ, CCL2, 3, and CXCL10; reduced numbers of infiltrating immune cells in the liver) following the induction of fulminant hepatitis using Concanavalin A injection. In that case, PTPRO KO was associated with increased PI3K/Akt activation and significantly elevated GSK3β-Ser9 phosphorylation [150].

Studying ischemic injury in an ischemia-reperfusion mouse model revealed that the respective WT mice exhibit significantly induced GSK3β activation (due to reduced Ser9 phosphorylation) together with strong inflammatory responses as reflected by enhanced leukocyte rolling/adhesion, numbers of circulating neutrophils, and TNF production. The injection of GSK3 inhibitor SB216763 during ischemia prevented the occurrence of inflammatory symptoms [151]. (Neuro)-inflammation is also a symptom observed upon hypoxic-ischemic injury. In a murine postnatal hypoxic-ischemic injury model, increased GSK3β activity and TNF/IL-6 expression were detected, which could be reduced by SB216763 [152]. 

Following induction of diabetes using streptozotocin, GSK3β activation by decreasing Ser9 phosphorylation was associated with increasing signs of inflammation (e.g., TNF, plasminogen activator inhibitor 1, and intracellular adhesion molecule 1 expression, 3-nitrotyrosine accumulation) in the liver of diabetic mice and (even more prominent) diabetic mice with zinc deficiency [153]. Under the same conditions, comparable results have been obtained in the liver of normal mice treated with a zinc chelator [153]. In contrast, SB216763-treated diabetic WT mice and cardiac-specific metallothionein-overexpressing transgenic mice (which are protected from diabetes-associated cardiomyopathy) showed neither GSK3β activation nor significant symptoms of cardiac inflammation [154]. In an alternative streptozotocin-induced diabetes model, elevated levels of GSK3β were accompanied with increased TNF, IL-1β, and IL-6 levels in the hippocampus. Pro-inflammatory cytokine expression could be ameliorated by application of *Boswellia serrate* extract, a polyphenol-rich substance also reducing hippocampal GSK3β expression [155]. Decreased GSK3β-Ser9 phosphorylation, increased TNF, IL-6, COX-2, and iNOS expression, and the abolishment of these effects by SB216763 can also be observed in the hippocampi of rats with diabetes induced by a combination of a high-fat diet and low streptozotocin concentrations [156].

An Alzheimer’s disease (AD) mouse model based on GSK3β overexpression is characterized by severe brain inflammation, e.g., increased numbers of activated microglia and enhanced TNF, IFN-γ, MIP-1α, -3α, and CCL2 (but also IL-10) expression [157]. In another AD model, in which the mice exhibit GSK3β hyperactivation and neuroinflammation, the application of tauroursodeoxycholic acid (an endogenous hydrophilic bile acid) led to the activation of Akt, increased GSK3β-Ser9 phosphorylation, reduced TNF expression, and decreased microglia activation [158].

These studies strongly suggest that active GSK3(β) is a potent driver of inflammation in vivo, whereas its inactivation has a mitigating influence. In consequence, the treatment with GSK3β-Ser9 phosphorylation-inducing substances, including a variety of natural products [21], generally dampens signs of (exaggerated) inflammation and tissue damage.

#### 3.1.3. Role of GSK3 During Bacterial Infections

During bacterial infections, GSK3 enzymatic activity may be modulated via toll-like receptors (TLR) and subsequent PI3K-Akt activation, effects implying an inhibition of GSK3 in response to bacteria and their compounds [159]. The impact of TLR signaling on GSK3 activity, however, is ambiguous, difficult to predict, and presumably dependent on the specific prevailing conditions (e.g., the cell type and timeframe of observation). Thus, GSK3-dependent pro- as well as anti-inflammatory responses have been reported following TLR activation.

For instance, Akt-mediated GSK3 inactivation following stimulation of TLR2, 4, 5, or 9 with appropriate agonists (lipoteichoic acid, LPS or synthetic *E. coli* lipid A, flagellin, and human CpG, respectively) significantly suppressed pro-inflammatory cytokine secretion and induced (TLR2-dependent) IL-10 production in human monocytes in a CREB- and CREB-binding protein (CBP)-dependent manner [90]. In LPS-treated murine macrophage-like RAW264.7 cells and primary murine macrophages, preceding TLR2 stimulation by recombinant leucine-responsive regulatory protein preincubation results in PI3K/Akt activation, GSK3β-Ser9 phosphorylation, reduced NF-κB activity/nuclear translocation, and suppression of pro-inflammatory IL-6 and -12 expression [160]. In LPS-challenged human monocytes, it was shown that Akt-dependent GSK3 inactivation may be supported by additional mTORC2-dependent activation of Akt as well as (mTORC1-dependent) activation of GSK3β-Ser9-targeting S6K [161]. The application of GSK3 inhibitors protected mice from endotoxin shock [90] and enhanced the survival of *Burkholderia pseudomallei*-infected mice [162]. The increase in IL-10 via CREB due to Akt-mediated GSK3β inactivation has also been demonstrated in murine macrophages in response to *Francisella tularensis* (FT) [163] and in murine and human macrophages in response to *Leishmania donovani* (LD) infection [164]. Furthermore, increased IL-10 and decreased IL-6 production due to Ser9-dependent GSK3β inactivation has been observed in PGN-treated primary murine peritoneal macrophages and RAW cells following EH application [148]. GSK3β-Ser9 phosphorylation has also been detected in FT-infected murine macrophages [163] and LPS- or LD infection-challenged RAW cells [165].

Interestingly, a concomitant application of the LD-derived TLR4 agonist β-1,4-galactose terminal glycoprotein (GP29) enhanced GSK3β activity, resulting in reduced CREB and increased NF-κB-p65 and AP-1-Jun/Fos phosphorylation, decreased IL-10 expression, and induced IL-12 and NO synthesis in LD-infected RAW cells [165]. Vaccination with GP29 promotes a protective immune effect in a murine visceral leishmaniasis model by restricting IL-10 and increasing the production of pro-inflammatory cytokines (TNF, IL-12, and IFN-γ), NO, and ROS [166]. Moreover, a GSK3-dependent upregulation of TNF and NO in response to *Streptococcus* infections via TLR2 has been observed in murine macrophages [125] and microglia [167].

#### 3.1.4. Role of GSK3 During Viral Infections

GSK3 also appears to be involved in the innate anti-viral immune response [159], though reports focusing on the effect of GSK3 on viral replication appear inconsistent and in part contradictory. This might be interpreted as different mechanistic approaches developed by viruses to subordinate the cellular machinery of the host or to escape the anti-viral activity of infected cells.

For instance, GSK3β has been identified as one of the host factors required for influenza A virus entry [168]. In human immunodeficiency virus (HIV-)1-infected T- and monocytic cell lines, upregulated GSK3β expression has been observed in both the cytoplasm and (to a lesser extent) the nucleus [169]. It has also been demonstrated that coxsackievirus B3 infection upregulated GSK3β levels in a mouse model [170] and increased the activity of GSK3β in HeLa cells, resulting in reduced β-catenin amounts, infection-associated cytopathic effects, and apoptosis as well as increased viral progeny release, effects that could be blocked by GSK3 inhibition [171]. Furthermore, GSK3β inhibition considerably reduces viral replication in HIV-1-infected macrophages or cell lines [169,172] and *Varicella zoster* virus-infected human MeWo melanoma cells [173]. In hepatitis C virus (HCV)-treated human hepatocarcinoma Huh7.5 cells, GSK3 inhibitors prevented the release of HCV virions, while viral replication was not affected [174]. Another group found that GSK3β (but not GSK3α) inhibition reduced both replication and viral particle production of HCV but not hepatitis E virus in Huh7.5 cells [175]. Moreover, GSK3-mediated phosphorylation appears to be involved in the destabilization of PHD finger protein 13, a host factor that (amongst other functions) is involved in repressing HIV-1 [176] and human cytomegalovirus gene expression [177]. Direct GSK3α/β-dependent phosphorylation of the coronavirus (CoV) nucelocapsid (N) has been described to be of importance for viral expansion, since GSK3 inhibition reduces both CoV-N phosphorylation and CoV replication [178].

On the other hand, the Karposi’s sarcoma-associated herpes virus-derived protein LANA is able to interact with GSK3β to favor nuclear GSK3β localization, and to increase both β-catenin [69,179] and Myc concentrations [180], which implies a LANA-dependent inactivation of GSK3β. Hepatitis B virus (HBV) replication is associated with increased GSK3β-Ser9 phosphorylation [181,182], indicating a beneficial effect of GSK3β inactivation for HBV replication. Mechanistically, anti-viral GSK3β effects may be associated in certain cases with its ability to enhance the anti-viral capacity of the zinc-finger anti-viral protein (an inhibitor of the replication of certain viruses) by sequential phosphorylation [183].

#### 3.1.5. GSK3 and Interferons

The expression of interferons, i.e., cytokines with anti-viral properties (amongst others), also affects GSK3 activity. For instance, stimulation with IFN-β leads to a Jak1-PI3K-Akt1-driven significant reduction of GSK3β activity, an accumulation of activated CREB in the nucleus, and the subsequent induction of anti-inflammatory IL-10 in human dendritic cells (DC) [88]. In murine DC, the LPS-driven increase in IL-12 and IL-23 can be limited, while IL-10 production can be intensified by IFN-β preincubation, an effect that is associated with enhancement of Akt and GSK3β-Ser9 phosphorylation [184].

IFN-γ, in contrast, increases GSK3 activity as reflected by hyper-phosphorylation of typical GSK3 targets, such as Tau protein [185]. IFN-γ-induced activation of GSK3 facilitates iNOS expression [186], decreases IL-10 production by suppressing CREB and AP-1 transactivation activity [145], and enhances LPS-induced pro-inflammatory IL-6 expression via signal transducer and activator of transcription (STAT) 3 [187]. In this context, an association of enzymatically active GSK3β with the IFN-γ receptor has been demonstrated [187]. Equivalently, IFN-γ-induced activation of GSK3β suppressed IL-10 production, resulting in a subsequent upregulation of TNF and NO synthesis [188]. Interestingly, IFN-γ-dependent activation of GSK3β appears to involve both phosphatase-mediated dephosphorylation at Ser9 and Pyk2-mediated phosphorylation at Tyr216 [188,189]. IFN-γ-induced activation of GSK3β can be counteracted by Ser9 phosphorylation-inducing agents as shown for the immunosuppressant mycophenolate [190].

#### 3.1.6. Clinical Application of GSK3 Inhibitors

Because of its remarkable impact on a variety of physiological key processes [191], GSK3 inhibition has been early regarded as a promising approach within the treatment of serious common diseases (also including inflammation or inflammatory components [17]), such as neurological [192] and cancerous [193] diseases. Though the initial high hopes have not been fully realized, GSK3 inhibitors are still under clinical investigation [194].

In several preclinical and clinical trials, the efficacy and safety of pharmacological GSK3 inhibitors for different clinical purposes are or have been addressed [194]. The most prominent GSK3 inhibiting agent is lithium, a non-selective metal cation whose precise inhibitory mechanism is still not fully elucidated. Lithium has been clinically applied for decades to patients with mood and bipolar disorders [195]. Thus, it is a well-characterized prevalent drug exhibiting an acceptable level of adverse effects [196], though in some elderly patients, toxic effects have been described [195]. Currently, the effect of lithium on Parkinson’s disease (NCT04273932, phase I), cognitive impairment (NCT03185208, phase IV) [197], and fracture healing (NCT02999022, phase II) [198] is being assessed.

Other common inhibitors studied are ATP analogs acting as reversible ATP competitors [195], such as LY2090314, a potent and relatively GSK3-selective inhibitor. Following promising preclinical and phase I clinical results in terms of pharmacokinetics, metabolism, excretion [199], and efficacy (in combination with pemetrexed and carboplatin) against advanced solid tumors [200], a phase II study showed no significant clinical effects of LY2090314 as a single agent in acute myeloid leukemia treatment, thus limiting its benefit [201]. Other components, such as AZD1080, were analyzed in phase I trials [202] but discontinued in development [195]. Chiefly, problems with this type of drug may be attributed to two major issues, i.e., a common lack of specificity combined with a frequently occurring toxicity [195]. However, even ATP competitors are still regarded as potential pharmaceutical agents. 9-ING-41, for instance, is a malmeimide-derived GSK3 inhibitor that initially showed potent anti-proliferative activity [203] and capacity to overcome chemoresistance [204] in different cancer cell lines. At the moment, the safety and efficacy of 9-ING-41 are being addressed in patients with hematologic malignancies and solid tumors in a phase I/II study (NCT03678883) [197,205].

Tideglusib, a thiadiazolidindione, is an oral administrable, non-ATP competitive inhibitor characterized by an irreversible inhibition of GSK3 [206]. Its therapeutic impact on AD [207] and progressive supranuclear palsy (PSP) [208] was assessed in a series of studies. Tideglusib was generally well tolerated, showed encouraging results in an AD pilot study [209], and appeared to reduce cerebral atrophy in a PSP subgroup in a phase II clinical trial [210]. However, no significant improvement in the Tideglusib-treated AD or PSP groups was observed in phase II clinical trials [207,208]. Following the completion of a phase II study (NCT02858908) assessing the safety, pharmacokinetics, and efficacy of Tideglusib in patients with myotonic dystrophy in 2018, a clinical phase II/III trial (NCT03692312) was announced for 2020 [197].

#### 3.1.7. Regulation of Transcriptional Systems and Associated Signaling Pathways

On the transcriptional level, GSK3-regulated effects are mediated via the activation or inactivation of prominent signaling pathways and TF, in particular NF-κB [137], AP-1 [211,212,213], and C/EBP [214], which are described below in detail. However, various further TF, such as STAT1 [189], 3, and 5 [215], or CREB [216], also contribute to the transmission of GSK3-dependent signaling.

##### NF-κB-associated signaling

NF-κB, a TF consisting of the subunits p105/p50 (i.e., NFKB1), p65 (RelA), and c-Rel (representing the canonical NF-κB pathway) as well as p100/p52 (NFKB2) and RelB (the non-canonical pathway) [217], is one of the most important transcriptional mediators transferring GSK3 activity into gene expression [218]. Initially, it has been demonstrated that TNF-induced NF-κB DNA binding activity and transactivation of NF-κB-dependent promoter constructs were significantly reduced in murine GSK3β KO fibroblasts, effects that could be reversed by transient transfection of rat GSK3β. Interestingly, under these circumstances, IκB degradation and nuclear p65 translocation were not affected [137]. GSK3 inhibition [219] or genetic deletion [220] also had no effect on IκBα degradation, IKK activation, or p65 translocation and it has been proposed that direct p65 phosphorylation may play a decisive role in that context (see below) [219]. In addition, during GSK3 inactivation, the increasing β-catenin levels appear to further enhance NF-κB suppression, since β-catenin has been shown to directly interact with protein complexes that include p65- and/or p50-containing NF-κB dimers and to reduce NF-κB DNA binding and subsequent functions (i.e., transactivation activity and target gene expression) in this way [221].

The constitutive DNA binding and transactivation activities of p65/p50 heterodimers observed in pancreatic cancer cells are positively regulated by both GSK3α and β, since GSK3 inhibition (including paralog-specific siRNA) results in reduced p65 phosphorylation, DNA binding, and NF-κB-dependent gene expression, while p50 homodimers seem to be unaffected. This mechanism appears to involve GSK3-dependent constitutive IKK activation and IκBα phosphorylation [222]. In TNF-stimulated WT endothelial cells, a rapid transient (i.e., 5–10 min following stimulation) activation of GSK3 has been observed, an effect associated with IKKβ activation, IκBα degradation, enhanced DNA binding activity of NF-κB, and the formation of a pro-inflammatory phenotype [223]. The influence of long-term TNF incubation on GSK3 activity, however, has not been comprehensively addressed yet (see also Section 3.2.4.). Other studies demonstrated that GSK3β activity contributes to the activation of several NF-κB-dependent genes following TNF stimulation by enhancing p65 DNA binding activity, while specific subsets of NF-κB-dependent genes (including IκBα) are not affected [220,224]. Vice versa, at the promoters of certain NF-κB-dependent genes that are suppressed under GSK3β inhibiting conditions, either decreased p65 or increased p50 amounts can be detected [224]. This suggests the existence of specific and differentially regulated NF-κB-dependent gene sets and a distinct role for GSK3β in the modulation of the NF-κB system(s). This may also be reflected by the identification of a set of growth factor-responsive genes that are suppressed in quiescent cells by GSK3-dependent inhibition of IKK and IκBα phosphorylation, whereas GSK3 inhibition (either by inhibitors or growth factor stimulation) leads to increased IKK activity, IκBα phosphorylation/degradation, nuclear p65/p50 translocation, and binding of p65 to the promoters of several (though not all) of these genes [225]. Thus, GSK3 activation appears to involve an activation of NF-κB and associated signaling molecules in general. However, since single studies report that overexpression of constitutively active GSK3β variants significantly reduced stimulus-induced IKK activation, IκBα degradation, NF-κB DNA binding activity, and/or NF-κB-dependent transcription [226,227], an excess of enzymatically active GSK3β also appears to be disadvantageous for the responsiveness of the NF-κB system.

Most of the NF-κB subunits also act as substrates of GSK3. GSK3-dependent phosphorylation has been described for p65 [219] at Thr254 [228], Ser276 [229,230], Ser468 [231,232] (or its murine equivalent Ser467 [230]) and Ser536 [222,233], p105 at Ser903 and 907 [234], p100 at Ser707 [235], and RelB at Ser552 (in the mouse; corresponds to human RelB-Ser573 [236]) [237]. Various functional consequences arise from GSK3-driven NF-κB phosphorylation. Phosphorylation of p65-Thr254 appears to be essential for the transactivation of certain genes as shown for type II collagen expression during chondrocyte differentiation [228]. An increase in GSK3β-Tyr216 phosphorylation and the Tyr216/Ser9 ratio was associated with enhanced p65-Ser276 phosphorylation, p65-CBP interaction, and pro-inflammatory cytokine expression (TNF, IL-1β) [229]. In unstimulated cells, phosphorylation of p65 at Ser468 inhibits its transactivation activity and reactivation may be performed by PP1-mediated dephosphorylation [231]. In stimulated cells, however, p65-Ser468 phosphorylation results in the increased expression of a subset of NF-κB-dependent pro-inflammatory genes, including monocyte chemoattractant protein-1 [230,232]. EGF-stimulated epithelial cells show an activation of the canonical NF-κB pathway as reflected by increased p65-Ser536 phosphorylation due to the activation of both IKKα and GSK3β [233].

The major effect of GSK3-dependent phosphorylation of p105 and p100 is the modulation of protein stability and precursor digestion. While the interaction with GSK3β stabilizes p105 in resting cells by reducing the rate of constitutive p105 to p50 degradation, GSK3β-mediated phosphorylation of p105 at Ser903 and Ser907 primes p105 for subsequent proteasomal degradation upon TNF stimulation [234]. In turn, formation of p50 from its precursor p105 by limited proteolysis is facilitated in the absence of GSK3 [234].

GSK3-dependent constitutive phosphorylation of p100 at Ser707 in the nucleus enables its association with the F-box protein Fbxw7α-containing ubiquitin ligase complex, resulting in its subsequent ubiquitination and proteasomal degradation. As a consequence, binding and inhibition of other NF-κB subunits (e.g., p52 and RelB) by p100 is abolished. Thus, the efficient clearance of nuclear p100 is a prerequisite for proper activation of the non-canonical NF-κB signaling pathway [235]. The proteasome-dependent generation of p52 from p100, however, appears to be performed in a GSK3-independent manner [235].

Following suitable stimulation, Ser552 phosphorylation of RelB by GSK3β leads to its efficient degradation, which can be prohibited using SB216763, siRNA, or enzymatically inactive GSK3β variants [237]. 

GSK3 also affects further factors of the NF-κB system. The stability of Bcl-3 (“B cell lymphoma 3”), an important interaction partner of p50 or p52 homodimers [238], is also negatively regulated by GSK3 via phosphorylation at Ser394 and Ser398, which induces subsequent ubiquitination and proteasomal degradation [239]. Moreover, binding and phosphorylation (especially at Ser8, 17, and 31) of NF-κB essential modifier (NEMO, aka IKKγ) by GSK3β is required for its stabilization and the proper mediation of NF-κB signaling. Artificial destabilization of NEMO by replacing the relevant GSK3 target residues by alanine is accompanied by increased Lys63-polyubiquitination of the remaining NEMO molecules, enhanced IKKα and β binding, and elevated constitutive IκBα degradation, effects that could be further intensified by GSK3 inhibition [240].

##### AP-1-Associated Signaling

The regulation of AP-1 is a crucial step in mediating the expression of a variety of GSK3-dependent genes, making AP-1 an important player within the GSK3-regulated transcriptional network [218]. In general, activation and activity of AP-1 and its subunits Jun, JunB, JunD, Fos, FosB, Fos-related antigen (Fra-)1, and Fra-2 are inhibited by active GSK3. Early, it was shown that GSK3 is able to phosphorylate the DNA binding domain within the C-terminus of Jun in vitro, thus reducing the DNA binding activity of AP-1 consisting of Jun homodimers [211], whereas PKC (i.e., PKCα, β1, β2, or γ)-mediated GSK3β inactivation reduces the negative regulatory Jun phosphorylation [91]. Once phosphorylated by GSK3, Jun family proteins may be ubiquitinated and subsequently degraded via the proteasomal pathway as shown for Jun [241] and JunB [242]. For JunB and D, a similar GSK3-driven regulation of DNA binding activity has been demonstrated [212,213]. However, since JunB/D and Fra-1/2 possess relatively weak transactivation domains, which may render them to AP-1 subunits with predominantly deactivating or repressing features, GSK3-driven regulation of AP-1 is often mediated via its activating subunits, such as Jun [218]. A good example for the regulatory interrelationship during inflammation is the repression of IL-10 via the inhibition of AP-1 (and CREB) following IFN-γ stimulation of primary human macrophages. In detail, IFN-γ induced the activation of GSK3, which was associated with a significant suppression of both basal and TLR2-induced fos mRNA and Jun protein levels, lower AP-1 DNA binding activity, and reduced expression of AP-1-dependent genes, such as IL-10 [145]. An alternative possibility to negatively regulate Jun/AP-1 activity is the suppression of JNK activating signal transduction as shown in *drosophila* [243].

Vice versa, the inhibition of GSK3 either using inhibitors [244] or in response to physiological stimulation, such as integrin receptor binding [245] or Fcε receptor I crosslinking [246], leads to an activation of AP-1. Under these circumstances, reduced phosphorylation and increased protein stability of Jun can be observed [244]. Moreover, Jun, Fra-1 [247], and Fos [248] have been shown to be induced in the presence of inactivated GSK3β or increased β-catenin levels. In diseases characterized by augmented amounts of Ser9-phosphorylated GSK3β levels, such as cholestatic liver disease, Jun levels are also increased [249]. However, in some cases, opposed effects have been observed. In murine BV-2 microglial cells, pretreatment with GSK3 inhibitor TWS119 reduced the LPS-dependent activating phosphorylation of JNK and Jun, AP-1 DNA binding activity, and AP-1-dependent reporter gene activation [250].

##### C/EBP-Associated Signaling

The C/EBP family (consisting of C/EBPα, β, γ, δ, ε, and ζ [44]) is also able to mediate GSK3-derived signals [214]. C/EBPα was early identified as a GSK3 substrate phosphorylated at Thr222 and Thr226 (corresponding to human Thr226/230 [236]), i.e., residues that are dephosphorylated by PP1 or 2a during GSK3 inhibition, e.g., in response to insulin [251]. Accordingly, in transgenic mice expressing inactivation-resistant GSK3α-Ser21Ala and GSK3β-Ser9Ala variants, C/EBPα-Thr222/226 phosphorylation was significantly enhanced, while total C/EBPα levels were comparable with the WT [252]. Furthermore, it has been described that GSK3 activity is able to induce conformational changes in C/EBPα [251]. However, since reduced Thr222/226 phosphorylation could not be detected in insulin-treated hepatocytes, this effect appears to be cell type specific [253]. Moreover, the functional role of these phospho-sites seems to be situation dependent, since no concrete regulatory influence has been observed by in vitro serine-to-alanine mutation in adipocytes [253], whereas these aa exchanges suppressed the induction of metallothionein 2A in murine hepatocytes [254].

An equivalent dephosphorylation following growth hormone-induced GSK3 inactivation has been observed for C/EBPβ and its isoforms liver-enriched activating protein (LAP, a TF possessing transactivating capacity) and liver-enriched inhibitory protein (LIP, an inhibitory factor lacking transactivation domains) in murine fibroblasts. In this case, interestingly, LAP DNA binding activity was increased, while LIP binding was decreased, at least at certain C/EBP binding sites, e.g., within the cfos promoter, an effect associated with increased promoter activity [255]. Thus, C/EBPβ may contribute to the activation of AP-1 under GSK3-inhibiting conditions (see Section 3.1.7.). In murine embryonic fibroblasts and macrophage-like cells, another phosphorylation site at Thr188 (human: Thr235 [236]) has been described that was required for the activation of certain genes, including C/EBPα, and is sensitive towards GSK3 inhibition [256,257]. Following stimulation of RAW cells with edema toxin (ET), the phosphorylation of C/EBPβ-Thr188 was enhanced and the association of both GSK3β and C/EBPβ with the tumor suppressor APC (“adenomatous polyposis coli”, a subunit of the β-catenin destruction complex [8]) was induced, suggesting that APC may facilitate GSK3-driven C/EBPβ-Thr188 phosphorylation [257]. Further, MAPK-phosphorylated Thr188 may act as a priming site for GSK3β-mediated phosphorylation of Ser184 and Ser179, i.e., phospho-residues favoring dimerization due to conformational changes and enhancing both DNA binding and transactivation activity [40,258]. Decreased C/EBPβ-Thr235 phosphorylation due to GSK3 inactivation was detected in TNF long-term-incubated monocytic cells in the presence of kenpaullone [259] as well as human monocyte-derived DC in response to platelet-activating factor [260]. In contrast, in IL-17-stimulated murine ST2 stromal cells, ERK-mediated priming phosphorylation at Thr188 and subsequent phosphorylation at Ser179 by GSK3 have been described as being repressive for certain C/EBP-dependent genes [261]. Equivalent results have been obtained in human umbilical vein endothelial cells (HUVEC) [262]. In differentiated murine preadipocytic 3T3-L1 cells, an O-linked β-N-acetylglucosamine modification at Ser180 or Ser181 prevented C/EBPβ-Thr188, -Ser184, and -Thr179 phosphorylation and decreased its DNA binding and transactivation activities [263]. In rat-derived osteoblasts, a corresponding GSK3β-dependent phosphorylation at C/EBPβ-Thr189, -Ser185, -Ser181, and -Ser177 was described. In this case, however, stimulation-induced and cGMP-dependent protein kinase (PKG)-dependent GSK3β inhibition was associated with C/EBPβ dephosphorylation, increased DNA binding activity, and enhanced C/EBPβ-dependent reporter gene expression [264], suggesting a differential regulation in the rat when compared to mice and humans. In murine microglial cells, LPS-induced nuclear translocation of C/EBPβ and δ was strongly enhanced in the presence of LiCl [265]. Moreover, murine C/EBPδ has been reported to be phosphorylated by GSK3β at Ser167 in response to pro-inflammatory cytokines, such as TNF and IL-1β, leading to enhanced C/EBPδ-dependent gene expression [266].

Under specific conditions, levels of C/EBP proteins appear to be (differentially) influenced by GSK3 activity. In prostate cancer cells, for instance, normally low C/EBPα protein (but not mRNA) levels massively increased in the presence of GSK3 inhibitors, while the higher C/EBPβ levels remained unchanged, suggesting that C/EBPα protein stability may be affected by GSK3 [267]. Inhibition of GSK3 activity by melatonin treatment led to downregulation of C/EBPβ and δ expression in human mesenchymal stem cells [268]. In RAW cells, C/EBPδ has been shown to be targeted for degradation by Fbxw7α following phosphorylation by GSK3β at Thr156 and (to a lesser extent) Ser160. GSK3β inhibition, in turn, either by inhibitors or LPS-induced Ser9 phosphorylation, enhanced C/EBPδ protein half-life [269]. In hepatocellular carcinoma cells, however, CEBPD promoter activation and C/EBPδ levels decreased in the presence of enhanced β-catenin amounts following GSK3 inactivation [270]. C/EBPζ, aka C/EBP homologous protein (acting as a pro-apoptotic TF), has been described to be upregulated in situations favoring GSK3α/β activation, such as endoplasmic reticulum (ER) stress [271]. Accordingly, expression of the constitutively active GSK3β-Ser9Ala variant induced C/EBPζ mRNA and protein levels in primary murine macrophages even under conditions antagonizing ER stress [272]

### 3.2. GSK3 and the Resolution of Inflammation

Negative modulation of GSK3 activity appears to be a crucial step during the regulatory events governing the resolution of inflammation, including the expression of pro-resolving cytokine profiles, clearance of apoptotic immune cells, and tissue repair/wound healing. However, under certain circumstances, GSK3 inhibition may also prohibit the termination of inflammation. In the following, the respective GSK3-associated mechanisms are discussed.

#### 3.2.1. GSK3 and the Expression of Pro-Resolving Cytokine Profiles

Data from a study focusing on spontaneous resolution of inflammation in a murine trinitrobenzene sulfonic acid-induced colitis model revealed that colitis-induced secretion of IL-13 led to the inactivation (i.e., Ser9 phosphorylation) of GSK3β via the STAT6-driven activation of p38. The following increase in nuclear CREB resulted in decreased pro-inflammatory NF-κB-p65 DNA binding as well as reduced IL-17 and IL-23 but increased IL-10 expression, i.e., a modified cytokine expression profile favoring amelioration of inflammation [94]. In THP-1 cells challenged with varying doses of LPS, GSK3β-dependent cytokine expression profiles were differentially modified, suggesting a dose-dependent impact of LPS on the programming of innate immune cells and the resolution of inflammation. In this model, very low LPS doses yielded decreased Akt activity and increased Pyk2-mediated Tyr216 phosphorylation (i.e., activation) of GSK3β, resulting in elevated expression levels of pro-inflammatory IL-6. At the transcriptional level, an upregulation of TF forkhead box protein O1 (FoxO1) and a downregulation of CREB protein amounts contribute to the realization of this effect. Following high dose treatment, THP-1 cells showed increased Akt activity (implying GSK3 inhibition), upregulated CREB and downregulated FoxO1 levels (including activating CREB-Ser133 and inhibiting FoxO1-Ser256 phosphorylation), and elevated levels of both IL-6 and anti-inflammatory IL-33 [129].

Interestingly, resolution of inflammation also comprises effects that are rather regarded as pro-inflammatory. For instance, the proper production of ROS has been implicated to be of importance, since p47^phox^ KO resulted in ROS deficiency-associated excessive acute inflammation in different mouse models (including p47^phox^ KO mice and macrophages) following LPS challenge. Mechanistically, this can be attributed to decreased IL-10 levels, which are caused by significantly increased GSK3β activation due to reduced PI3K-Akt-dependent GSK3β-Ser9 phosphorylation in the absence of p47^phox^ [273]. Thus, GSK3 inhibition generally appears to support the resolution of inflammatory processes. Consequently, the application of GSK3 inhibitors (e.g., SB216763, SB415286, TDZD-8 [274], indirubin-3′-monoxime [275], or lithium [145,276]) alleviates symptoms of systemic inflammation in murine and rat inflammatory disease models [121] (see Section 3.1.2.) and equivalent results have been obtained in human cell culture approaches [223].

#### 3.2.2. GSK3 and the Clearance of Apoptotic Immune Cells

Clearance of apoptotic immune cells by macrophage-mediated efferocytosis is a key feature of resolving inflammation [277]. In PMA-differentiated human premonocytic THP-1 cells, treatment with the anti-inflammatory agent lipoxin A_4_ leads to increased phagocytosis of apoptotic polymorphonuclear neutrophils and lymphocytes in the dependence of PKCζ-mediated GSK3β-Ser9 phosphorylation [278]. Using primary human monocyte-derived macrophages, another study showed that during efferocytosis, GSK3β inhibition may be supported by miR-21 expression via the miR-21-induced silencing of phosphatase PTEN (“phosphatase and tensin homolog”), since this effect enables the activation of PI3K and GSK3-Ser9 phosphorylation [279].

#### 3.2.3. GSK3 and Tissue Repair

In an in vitro airway injury and repair model, it was demonstrated that scratching-induced injury of a bronchial epithelial cell monolayer caused (PKC-dependent) GSK3β-Ser9 phosphorylation, accumulation and nuclear translocation of β-catenin, and increased cyclin D1 expression. These pro-proliferative events supported tissue repair, a process also contributing to the termination of inflammation [280]. An equivalent regulation of GSK3 has been observed in a wounding model using IEC-18 rat epithelial cells [281]. Consistently, wound healing was supported by overexpression of β-catenin or substances inducing GSK3β-Ser9 phosphorylation, such as lucidone [282], while GSK3β overexpression or PKC inhibition had the opposite effect [280]. Interestingly, the complete absence of GSK3β seems to have an adverse effect, since GSK3β KO murine embryonic fibroblasts exhibited reduced restitution in response to scrape wounding [281].

#### 3.2.4. Inhibition of GSK3 and the Perpetuation of Inflammation

In certain situations, GSK3β inactivation has proven to counteract the termination of inflammation. The elimination of neutrophils, for instance, which represents another key module of fading inflammation [283], is significantly impaired in patients with severe injuries showing resistance towards intrinsic neutrophil apoptosis [284]. At the molecular level, reduced GSK3β enzymatic activity prevents the phosphorylation and subsequent ubiquitination/degradation of the anti-apoptotic protein Mcl-1 (“myeloid cell leukemia 1”). Thus, the stabilization and accumulation of Mcl-1 results in the suppression of pro-apoptotic events and a prolonged neutrophil lifespan [284]. Another example is the IL-17A-induced promotion of pulmonary fibrosis. Here, IL-17A stimulation results in PI3K-dependent inactivation of GSK3β, inhibition of GSK3β-Bcl-2 binding, reduced Bcl-2 phosphorylation and ubiquitination, and increased Bcl-2 levels in murine MLE-12 alveolar epithelial cells. Pathophysiologically, this leads to reduced autophagy and retarded resolution of inflammation, which enhances pulmonary fibrosis [285].

The development of different forms of tolerance towards the sustained stimulation with various activating agents is also regarded as an event contributing to the resolution of inflammation. In this context, the term tolerance describes a physiological phenomenon in which a pretreatment of organisms or specific cell types (e.g., monocyte/macrophages or hepatocytes) with a specific activating stimulus over a certain time leads to the resistance towards further stimulation with the same (or, in the case of cross-tolerance, another) substance. In its pure form, tolerance is mostly expressed as TNF, LPS, or TNF/LPS (cross-)tolerance [286] and reflected by the repression of immunologically relevant genes, such as IL-8 [287] or Il-6 [288]. TNF tolerance may occur in two forms, i.e., absolute tolerance (in which gene expression is blocked) and induction tolerance (in which gene expression is not further inducible). Low-dose preincubation predominantly results in absolute tolerance, whereas high-dose preincubation induces both absolute and induction tolerance [289]. Amongst other mechanisms, the development of low-dose TNF tolerance as well as TNF-induced cross-tolerance towards LPS depends on the activity of GSK3 [286], since GSK3 remained in an active state during TNF preincubation [288], while pharmacological inhibition or genetic deletion of GSK3 are able to reverse the formation of these forms of tolerance [288,289]. Mechanistically, GSK3 inhibition appears to counteract several molecular effects observed in low-dose tolerized cells, especially the increased phosphorylation of NF-κB-p65 at the inhibiting site Ser468 and the reduced phosphorylation at the activating site Ser536 [289]. The latter can be traced to direct association of C/EBPβ with p65 in tolerant cells [290]. It has been further shown that a GSK3 inhibitor can suppress the rapid resynthesis and nuclear accumulation of IκBα that occurs during LPS stimulation following TNF preincubation, suggesting that GSK3 is involved in the IκBα-mediated restriction of NF-κB signaling under these conditions [288]. In addition, GSK3 inhibition in TNF-tolerized cells also results in increased binding of p65 at the promoters of tolerizable genes (e.g., IL-6), reduced expression of A20 (a negative regulator of NF-κB signaling, e.g., during TNF incubation [291]), and enhanced chromatin accessibility [288]. Recently, it has further been demonstrated that numerous proteins differentially phosphorylated under conditions inducing high-dose TNF tolerance are potential GSK3 targets [259]. However, further analyses have to elucidate whether additional GSK3-dependent mechanisms are involved in the complex regulation of tolerance formation and the termination of pro-inflammatory signaling.

## 4. Concluding Remarks

As shown in this review, GSK3 is a versatile kinase targeting a variety of substrates. Thus, GSK3 controls a plethora of cellular functions at various mechanistic levels by orchestrating numerous molecular events. With respect to inflammation, GSK3 acts as a potent and multifunctional regulator of both pro- and anti-inflammatory effects. Among both paralogs, GSK3β appears to be the more influential kinase in terms of imunoregulatory action. Its specific effect, however, appears to depend on the respective cellular and molecular conditions. In general, active GSK3 involves a strong pro-inflammatory component by supporting the execution of multifaceted pro-inflammatory events. Conversely, GSK3 inhibition, either following physiological Ser9 phosphorylation or via pharmacological approaches, predominantly contributes to the amelioration of inflammation (as summarized in Table 1). The latter also includes the termination of pro-inflammatory signaling and the induction of (cross-)tolerance. Remarkably, several exceptions from this principle have been described. This janiform and to some extent contradictory nature is part of the mystery surrounding GSK3. During inflammation, modulation of GSK3(β) activity may affect multiple regulatory levels. These include enzymatic activity, signal transduction, transcription and translation, as well as localization and stability. Thus, due to its two-faced nature and its eclectic regulatory possibilities, GSK3 can be regarded as a key factor steering and balancing the initiation, progress, and resolution of inflammation. In consequence, a better understanding of its complex modulatory role during inflammatory processes may represent a promising opportunity to specify and modify different stages of inflammatory diseases.

## Figures and Tables

**Table 1:** Molecular events regulated by GSK3 that mediate pro- or anti-inflammatory effects.

Predominantly Pro-Inflammatory Effects. (Due to Active GSK3)	Predominantly Anti-Inflammatory Effects (Due to GSK3 Inhibition)
NF-κB DNA binding activity ↑	NF-κB DNA binding activity ↓
NF-κB transactivation activity ↑ (pro-inflammatory genes)	NF-κB transactivation activity ↓ (pro-inflammatory genes)
p65 phosphorylation (Thr254, Ser276, Ser536) ↑	p65 phosphorylation (Ser468) ↑
p105 phosphorylation (Ser903, Ser907)* ↑	p105 stability ↑
p100 phosphorylation (Ser707)* ↑	p100 stability ↑
NEMO phosphorylation (Ser8, Ser17, Ser31) ↑	-
RelB phosphorylation (Ser552)* ↑	RelB stability ↑
Bcl-3 phosphorylation (Ser394, Ser398)* ↑	Bcl-3 stability ↑
Jun/Fos phosphorylation ↑	Jun/Fos phosphorylation ↓
AP-1 DNA binding activity ↓	AP-1 DNA binding activity ↑
C/EBPβ-LIP DNA binding activity ↑ (anti-inflammatory genes)	C/EBPβ-LAP DNA binding activity ↑ (anti-inflammatory genes)
C/EBPβ transactivation activity ↓ (anti-inflammatory genes)	C/EBPβ transactivation activity ↑ (anti-inflammatory genes)
C/EBPβ phosphorylation (Thr188, Ser184, Ser179) ↑	C/EBPβ phosphorylation (Thr188, Ser184, Ser179) ↓
CREB activity ↓	CREB activity ↑

* leads to subsequent degradation. ↑, increased; ↓, decreased.

## Abbreviations

**Abbr.**	**Terms**
aa	amino acid
AD	Alzheimer’s Disease
AMPK	AMP-activated protein kinase
AP	activator protein
APC	adenomatous polyposis coli
Bcl	B cell lymphoma
BM	bone marrow
β-TrCP	F-box containing E3 ligase β-transducin repeat-containing protein
C/EBP	CCAAT/enhancer binding protein
cAMP	cyclic adenosine monophosphate
CBP	CREB binding protein
CCL	C-C-motif chemokine ligand
CIA	collagen-induced arthritis
CoV	Coronavirus
CoV-N	Coronavirus nucelocapsid
COX	cyclooxygenase
CREB	cAMP-responsive element binding protein
CXCL	C-X-C-motif chemokine ligand
EH	ephedrine hydrochloride
ER	endoplasmic reticulum
ERK	extracellular signal-regulated kinase
ET	edema toxin
Fox	forkhead box protein
Fra	Fos-related antigen
FT	Francisella tularensis
GP29	β-1,4-galactose terminal glycoprotein
GS	glycogen synthase
GSK	glycogen synthase kinase
HBV	Hepatitis B virus
HCV	Hepatitis C virus
HIV	Human immunodeficiency virus
HUVEC	human umbilical vein endothelial cells
Hsp90	heat shock protein 90
IFN	interferon
IKK	IκB kinase
IL	interleukin
iNOS	inducible NO synthase
IRF	interferon response factor
IκB	inhibitor of κB
JNK	Jun N-terminal kinase
KO	knock-out
LANA	latency-associated nuclear antigen
LAP	liver-enriched activating protein
LD	Leishmania donovani
LIP	liver-enriched inhibitory protein
LPS	lipopolysaccharide
MAPK	mitogen-activated protein kinase
Mcl	myeloid cell leukemia
MIP	macrophage inflammatory protein
MMP	matrix metalloprotease
mTORC	mechanistic target of rapamycin complex
Myb	myoblastosis
Myc	avian myelocytomatosis viral oncogene homolog (Myc)
NEMO	NF-κB essential modifier
NF-κB	nuclear factor κB
NLS	nuclear localization sequence
NO	nitric oxide
P70-S6K	p70 ribosomal protein S6 kinase
P90-RSK	p90 ribosomal protein S6 kinase
PGN	peptidoglycan
PI3K	phosphatidylinositol-3-kinase
PKA	protein kinase A
PKB/Akt	protein kinase B
PKC	protein kinase C
PKG	cGMP-dependent protein kinase
PLIN	perilipin
PP	protein phosphatase
PSP	progressive supranuclear palsy
PTEN	phosphatase and tensin homolog
PTPRO	protein tyrosine phosphatase receptor type O
ROS	reactive oxygen species
SIRT	sirtuin
STAT	signal transducer and activator of transcription
TBK	TANK-binding kinase
TF	transcription factor
TGF	transforming growth factor
TLR	toll-like receptor
TNF	tumor necrosis factor
Tst	Tuberculin skin test reactivity, absence of
UTR	untranslated region
Wnt	wingless-type MMTV integration site family member
WT	wildtype
